# Development of Chimera AMP–Endolysin with Wider Spectra Against Gram-Negative Bacteria Using High-Throughput Assay

**DOI:** 10.3390/v17020200

**Published:** 2025-01-30

**Authors:** Masato Kogawa, Takuya Yoda, Ayumi Matsuhashi, Ai Matsushita, Yoshiki Otsuka, Shohei Shibagaki, Masahito Hosokawa, Soichiro Tsuda

**Affiliations:** 1 bitBiome, Inc., 513 Wasedatsurumaki-Cho, Shinjuku-Ku 162-0041, Tokyo, Japan; 2 Graduate School of Advanced Science and Engineering, Waseda University, 2-2 Wakamatsu-Cho, Shinjuku-Ku 162-8480, Tokyo, Japan; 3 Computational Bio Big-Data Open Innovation Laboratory, National Institute of Advanced Industrial Science and Technology, 3-4-1 Okubo, Shinjuku-Ku 169-8555, Tokyo, Japan; 4 Institute for Advanced Research of Biosystem Dynamics, Waseda Research Institute for Science and Engineering, 3-4-1 Okubo, Shinjuku-Ku 169-8555, Tokyo, Japan; 5 Research Organization for Nano and Life Innovation, Waseda University, 513 Wasedatsurumaki-Cho, Shinjuku-Ku 162-0041, Tokyo, Japan

**Keywords:** endolysin, gram-negative bacteria, high-throughput screening

## Abstract

Bacteriophage-derived endolysins are being developed as an alternative to antimicrobials. The development of endolysins against Gram-negative bacteria requires the discovery of effective endolysins against the target species and the capability to penetrate the outer membrane of bacteria by endolysin. Here, we propose an efficient endolysin development approach that combines a data-driven endolysin search utilizing bacterial genomes with high-throughput laboratory assays. As a proof of concept, we analyzed endolysin genes detected in 273 bacterial genomes of *Acinetobacter*, *Pseudomonas*, and *Escherichia*. Firstly, we conducted assays of 192 recombinants of endolysin genes obtained through in silico search from bacterial genomes and identified natural endolysins degrading peptidoglycan of *Acinetobacter baumannii*. Then, we performed high-throughput screening against Gram-negative bacteria for hundreds of chimera AMP–endolysins, natural endolysin conjugated with antimicrobial peptide. As a result, we obtained four chimera AMP–endolysins against A. baumannii, which demonstrated the minimum inhibitory concentration ranging from 4 to 8 μg/mL. Moreover, we assessed the antimicrobial spectra of these chimera AMP–endolysins, validating that two endolysins exhibited antimicrobial efficacy against *Pseudomonas aeruginosa* and *Escherichia coli* with <32 μg/mL of concentration. This endolysin development approach can be applied to other Gram-negative bacterial targets and is expected to facilitate the acquisition of effective novel endolysins.

## 1. Introduction

In the past few decades, antibiotics have been widely used for the treatment of bacterial infections. However, in recent years, the emergence of drug-resistant bacteria has become a global healthcare threat [1,2]. In particular, there are limited treatments against multidrug-resistant bacteria known as ESKAPE pathogens (*Enterococcus faecium*, *Staphylococcus aureus*, *Klebsiella pneumoniae*, *Acinetobacter baumannii*, *Pseudomonas aeruginosa*, and *Enterobacter* species) which often cause severe infections. Thus, there is an urgent need for new approaches to treat drug-resistant bacteria [3,4,5].

Endolysins, which are peptidoglycan hydrolases derived from bacteriophages, are considered promising alternatives to antibiotics. Endolysins degrade the cell walls of host bacteria from within and facilitate phage release during the lytic cycle [6,7,8]. Endolysins typically consist of one or more cell wall-binding domains and enzymatic activity domains responsible for peptidoglycan degradation [9,10]. Due to the substrate selectivity of both domains, each endolysin exhibits distinct antimicrobial activity against specific bacterial species, making them potential candidates for highly selective antimicrobial agents. Many studies have been reported on obtaining endolysins mainly from isolated phage against host bacteria from 50 years ago, and the number of studies continues to grow [11,12,13]. Moreover, several reports have highlighted the development of chimera AMP–endolysins with altered antimicrobial spectra and increased activity through protein engineering techniques such as domain shuffling [14,15,16,17].

When using endolysins as antimicrobial agents, they need to reach the bacterial cell wall from the outside, unlike their original action inside the host cell in phage lytic cycles [18] Gram-positive bacteria can be rapidly lysed upon endolysin administration because their peptidoglycan layer is located at the outermost layer of bacterial membrane. In contrast, the lytic activity of endolysins against Gram-negative bacteria is significantly reduced because their peptidoglycan layer is covered by lipopolysaccharides (the outer membrane). The ability of endolysins to penetrate the outer membrane of gram-negative bacteria is important to exhibit antimicrobial activity [19]. Therefore, artificial endolysins like artilysin have been developed by conjugating charged amino acid residues like antimicrobial peptides (AMPs) to the end of the endolysins [20,21,22,23]. This enhances their antimicrobial ability by improving their outer membrane-penetrating ability. Because cationic peptides destabilizing the outer membrane exhibit a lower species specificity and the peptidoglycans of Gram-negative bacteria are less diverse than those of Gram-negative bacteria, artilysins targeting Gram-negative bacteria tend to have broader antimicrobial spectra compared to those targeting Gram-positive bacteria [24].

In this study, we aimed to develop multiple endolysins against Gram-negative bacteria, using a data-driven approach to design engineered endolysins with different antimicrobial spectra. We applied a high-throughput screening method to evaluate the resulting endolysins. Firstly, we conducted assays of 192 recombinants of endolysin genes obtained through an in silico search from bacterial genomes, determining natural endolysins against *A. baumannii*. Subsequently, we performed assays of 744 recombinants of chimera AMP–endolysins, which were created by random conjugation of the natural endolysins and AMPs. As a result, we obtained four chimera AMP–endolysins against *A. baumannii*. Furthermore, we evaluated the antimicrobial spectra of these chimera AMP–endolysins, confirming that some endolysins exhibited antimicrobial activity against *P. aeruginosa* and *E. coli*, indicating broad antimicrobial spectra. Our approach holds the potential for the efficient development of endolysins with different characteristics depending on their intended applications.

## 2. Materials and Methods

### 2.1. Bacterial Strains, Media, and Growth Conditions

The bacterial strains used in this study were as follows (Table S4): *Acinetobacter baumannii* str. NBRC 110489 (National Institute of Technology and Evaluation, NITE), *Pseudomonas aeruginosa* str. NBRC 12582 (NITE), *Escherichia coli* str. ATCC 10798, *Bacillus subtilis* str. ATCC 6633, *Enterobacter cloacae* str. NBRC 13535 (NITE), *Enterococcus faecalis* str. NBRC 100482 (NITE), *Fusobacterium nucleatum* str. JCM 8532 (JCM), *Klebsiella aerogenes* str. NBRC 13534 (NITE), *Klebsiella pneumoniae* str. BAA-1705 (ATCC), *Staphylococcus aureus* str. NBRC 100910 (NITE), and *Streptococcus gallolyticus* str. ATCC BAA-2069 (BEI resources). The following media were used for the culture of the bacterial strains: LB broth, Miller (Nacalai Tesque, kyoto Japan) for *A. baumannii*, *E. coli* and *P. aeruginosa*, brain heart infusion medium (Sigma-Aldrich, St. Louis, MO, USA) for *B. subtilis* and *F. nucleatum*, Lactobacilli MRS broth (BD) for *E. faecalis*, NBRC medium 802 (hipolypepton 10 g, yeast extract 2 g, MgSO_4_·7H_2_O 1 g in 1 L distilled water) for *E. cloacae* and *K. aerogenes*, tryptic soy broth (TSB) (Becton Dickinson, Franklin Lakes, NJ, USA) for *K. pneumoniae* and *S. gallolyticus*, and TSB with 7.5% NaCl (Nacalai Tesque, Japan) for *S. aureus*. *B. subtilis* was incubated at 30 °C, and the other strains were incubated at 37 °C. The AnaeroPack System (Mitsubishi Gas Chemical, Tokyo, Japan) was used for *F. nucleatum*.

### 2.2. DNA Extraction and Genome Sequencing

Genomic sequences of *Acinetobacter*, *Pseudomonas*, and *Escherichia* from bacterial isolate or single bacterial cells were collected to use for in silico search of endolysins. To acquire bacterial isolate genomic sequences, DNAs of the 96 isolates of *Acinetobacter* bacterium and 96 isolates of *Pseudomonas* bacterium were extracted using the DNeasy 96 Blood & Tissue kit (Qiagen, Hilden, Germany). Sequencing libraries were prepared using the QIAseq FX DNA Library Kit (Qiagen, Japan). Each library was sequenced using the Illumina NextSeq2000 2 × 150 bp configuration (Illumina, San Diego, CA, USA). In addition to the genomic data of the isolates, single-cell genomic data obtained in previous research were also used in the endolysin search [25]. Finally, 273 genomes were collected (116 *Acinetobacter* genomes, 144 *Pseudomonas* genomes, 13 *Escherichia* genomes).

### 2.3. Identification of Endolysin Genes from Microbial Genome Data

Endolysin genes were searched from the collected bacterial genomic sequences by the same in silico pipeline as in our previous studies [26]. Prophage sequences in bacterial genomes were predicted by PhageBoost 0.1.7 (options: -cs 200) [27]. Then, genes on the prophage regions were called by PHANOTATE 1.5.0 [28], and endolysin-like genes were collected by DIAMOND 2.0.5 search using endolysin genes in the RefSeq database [29]. Finally, Pfam domains on the collected genes were predicted by InterProScan 5.54-87 (options: -appl Pfam) [30], and endolysin candidates were selected based on the presence of the known endolysin-associated domains [31]. Unless otherwise noted, bioinformatic tools were conducted with the default option.

### 2.4. Construction of the Endolysin Library

Endolysin gene fragments were acquired by PCR from the extracted genomic DNA of bacterial isolates or single-cell MDA (multiple displacement amplification) products, which is the same way as in previous studies [32]. PCR amplicons were purified with the Wizard SV Gel and PCR Clean-Up System kit (Promega, Japan). Then, PCR amplicons and linearized pET17b vectors were assembled with NEBuilder HiFi DNA Assembly (NEB, Japan). The endolysin libraries were transformed into competent *E. coli* BL21(DE3)pLysS (Promega, Madison, WI, USA) by heat shock, and the recombinants were selected on LB agar plates containing 100 μg/mL ampicillin and 20 μg/mL chloramphenicol. In the construction of a chimeric endolysin library, PCR products of antimicrobial peptide genes and endolysin genes, which can be ligated by the linker sequence as N′-[AMP]-GAGAGA-[Endolysin]-His6-C′, were assembled into pET17b vector.

### 2.5. Endolysin Expression and Lysate Preparation

Single colonies of recombinants were picked into each well of 96-well deep plate filled with 500 µL of LB medium containing 100 μg/mL ampicillin and 20 μg/mL chloramphenicol. After incubation for 16 h at 37 °C, the 25 µL of culture was transferred into 500 µL of LB medium supplemented with the Overnight Express Autoinduction System 1 (Novagen, Japan). Then, cultures of recombinants were continuously incubated for 5 h at 37 °C and 16 h at 16 °C with shaking at 1000 rpm. After the centrifugation at 2000× *g* for 20 min, the cell pellets were treated with chloroform vapor for 1 h at room temperature to lyse the cells. Then, the residual chloroform was removed by incubation of the plate at 37 °C for 30 min. Finally, the pellets were suspended with 500 µL of HEPES buffer (20 mM HEPES-NaOH, 150 mM NaCl, pH 7.4) containing 0.4 U/mL DNase I (Takara, Japan) and incubated for 1 h at 30 °C. The lysate was stored at 4 °C.

### 2.6. Plate Lysis Assay

Plate lysis assays were performed with a modified method from a previous study [33]. Cultures of *A. baumannii* and *P. aeruginosa* in 100 mL media were autoclaved, and sterilized cells were collected by centrifugation. The cell pellets were suspended in three ml of HEPES buffer. Then, the peptidoglycan agar plate was made from a mixture of the cell suspension and 100 mL of HEPES buffer containing 1.5 % agarose. Four µL of lysate of recombinants was dropped onto the plate and lysis was observed by transparent area after incubation for 1 h at 37 °C. The transparent area was measured using ImageJ with the following commands: “Subtract Background” -> “Auto Local Threshold” with MidGrey method -> “Fill Holes” -> “Analyze Particles” [34].

### 2.7. Growth Inhibition Assay

*A. baumannii* were cultured overnight and diluted to OD_600_ = 0.1 with 2× BD BBL Mueller Hinton II Broth (BD) with 100 mM NaCl. The diluted cell suspension and the lysate of recombinants of 50 µL each were mixed and incubated for 16 h at 37 °C. Cell growth was monitored by measuring OD_600_ by Infinite 200 PRO M Nano+ (Tecan). Then, the normalized bacterial growth was calculated according to the following equation:

$$Normalized\;Growth=\frac{{OD}_{sample}-{OD}_{blank}}{{OD}_{control}-{OD}_{blank}}$$

The samples exhibiting <80% cell growth compared to the control sample (*A. baumannii* treated with lysate of the recombinant containing empty pET17b vector) were selected as growth-inhibiting endolysin.

### 2.8. Expression and Purification of Endolysins

The recombinants were cultured at 37 °C until the OD_600_ value reached 0.4–0.6, and IPTG was added to a final concentration of 0.5 mM. After overnight incubation at 16 °C, bacterial cells were harvested from 50 mL of culture and lysed with xTractor™ buffer (Clontech, Shiga, Japan) containing 2.5 U/mL DNase I (Takara Bio, Shiga, Japan) on ice. Then, endolysin protein was purified with the Capturem™ His-Tagged Purification Maxiprep Kit (Clontech, Japan) according to product protocols. After dialysis with PBS, the size of the purified protein was evaluated by SDS-PAGE with e-PAGEL (1020L, ATTO, Tokyo, Japan).

### 2.9. Endolysin Activity Assay

MIC values were determined using the methods based on the broth microdilution method for MIC determination, in which the CLSI guideline is M07-A9. The overnight culture of bacteria was inoculated into the appropriate growth medium and incubated at 37 °C until the OD_600_ reached 0.8. The bacterial cells were collected and suspended in a doubled concentration of cation-adjusted Mueller–Hinton broth (ca-MHB, BD) to achieve an OD_600_ value of 0.1. The cell suspension was diluted 100-fold and exposed to endolysins or antibiotics in a series of two-fold serial dilutions in the 96-well microtiter plate. After 18 h of incubation at 37 °C, the turbidity of the cell suspension was measured. The MIC was defined as the lowest drug concentration that kept the OD_600_ of cell suspension below 0.2, based on the turbidity of the suspension in which no bacterial growth was visually observed. The normalized bacterial growth was calculated according to the equation described in Section 2.7.

A time–kill curve was evaluated by kinetic absorbance of the bacterial cell suspensions for short-term. The bacterial cells in the overnight culture were collected and resuspended into the reaction buffer (20 mM Tris pH 7.5, 150 mM NaCl, 0.9 mM MgCl_2_, 2.5 mM CaCl_2_). This cell suspension was adjusted to OD_600_ = 0.8 with the reaction buffer, and 90 µL of cell suspension and 10 µL of endolysin solution at the concentration of 256 μg/mL were mixed. The measurement of the OD_600_ immediately started and was recorded every minute for 30 min at room temperature. Then, the OD_600_ at n minutes was normalized according to the following equation:

$$Normalized\;{OD}_{sample\_n}=\frac{{OD}_{sample\_0}-{OD}_{sample\_n}}{{OD}_{control\_0}-{OD}_{control\_n}}$$

### 2.10. Cytotoxicity Assay

The cytotoxicity effect of endolysins was evaluated with the Caco-2 cell line. A Caco-2 cell suspension was prepared with the DMEM medium (500 mL of Advanced DMEM/F12 (Gibco, Shinagawa-ku,Japan), 100 mL of fetal bovine serum, 6 mL of penicillin–streptomycin solution (Fujifilm, Tokyo,Japan), and 6 mL of GlutaMax (Gibco, Japan)) at a concentration of 1 × 10^5^ cells/mL. The cell suspension of 100 µL was added to each well of the 96-well microtiter plate and incubated for 24 h at 37 °C in 5% CO_2_. After the incubation, the cells were exposed with endolysins or compounds at a concentration of 50 or 100 μg/mL for 24 h at 37 °C in 5% CO_2_. Then, 10 µL of Cell Counting Kit-8 reagent (MedChemExpress Ltd., Shibuya-ku, Japan) was added to each well. After incubation for 4 h, the OD450 was measured to evaluate cell viability.

### 2.11. Biofilm Assay

The biofilm formation of *P. aeruginosa* was performed using methods from a previous study [35]. The overnight culture of *P. aeruginosa* was adjusted to OD600 = 0.001 with LB broth and statically incubated for 24 h at 37 °C in the 96-well microtiter plate. The culture was removed from the plate by decanting and the biofilm was gently washed three times with PBS. Then, an endolysin solution and an antibiotic solution were prepared with the reaction buffer (20 mM Tris pH 7.5, 150 mM NaCl, 0.9 mM MgCl_2_, 2.5 mM CaCl_2_), and the biofilm was exposed to endolysin or antibiotics at the concentration of 10 μg/mL for 24 h at 37 °C. The reaction solution was removed from the plate by decanting and the remaining biofilm was gently washed with PBS. The biofilm was stained with 0.05% crystal violet stain (20% methanol including 0.5 mg/mL crystal violet) for 15 min and washed with PBS after removal of the stain. Finally, the stained biofilm was suspended with 33% acetic acid, and OD_590_ of the suspension was measured.

## 3. Results

### 3.1. Identification of Endolysin Genes from Genomes of Gram-Negative Bacteria

**Figure 1:**
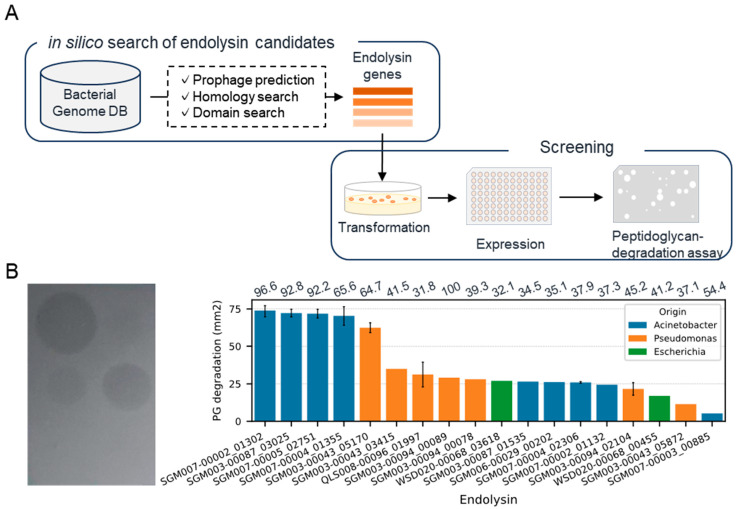
Discovery of natural endolysins against *A. baumannii.* (**A**) Workflow for the identification of endolysin against *A. baumannii*. (**B**) Representative image (left) and summary (right) of plate lysis assay using a lysate of recombinant expressing endolysins. The bar represents the area where the turbidity of the agar plate decreased. Error bars were only displayed for genes evaluated by lysates from multiple clones. The numbers above each bar exhibit identity (%) toward top hit endolysin in PhaLP.

We conducted an in silico search of endolysin genes from the genomes of *Acinetobacter*, *Pseudomonas*, and *Escherichia*, which are related to drug-resistant Gram-negative bacteria (Figure 1A). We collected bacterial genomes (116 *Acinetobacter*, 144 *Pseudomonas*, 13 *Escherichia*) from our database containing genomic sequences of human-associated bacteria obtained through the genome analysis of isolates or single-cell genome sequencing [25]. As a result of an endolysin gene search within the prophage regions of the bacterial genome, we detected 701 candidate genes (367, 315, and 19 genes from *Acinetobacter*, *Pseudomonas*, and *Escherichia* bacterial genomes, respectively). Subsequently, we constructed recombinants of 52 randomly selected genes expressed in *Escherichia coli* BL21(DE3) and evaluated their ability to lyse *A. baumannii* (Table S1). As a result of the plate lysis assay toward 192 recombinants, 18 recombinants demonstrated lytic activity, suggesting their potential antimicrobial activity against *A. baumannii* (Figure 1B). Among these 18 recombinants, five exhibited particularly significant lytic activity. Notably, two of these were obtained from different bacterial species and genera, *Acinetobacter junii* and *Pseudomonas mendocina*, hinting at endolysins with antimicrobial activity across a broad range of bacterial species. On the other hand, in the plate lysis assay with *P. aeruginosa*, over 95% of the recombinants displayed degradation (Table S2). Based on these results, we decided to use all 52 endolysin genes for the next experiment, the construction of chimera AMP–endolysins.

### 3.2. Screening of Chimeric Endolysin Libraries

**Figure 2:**
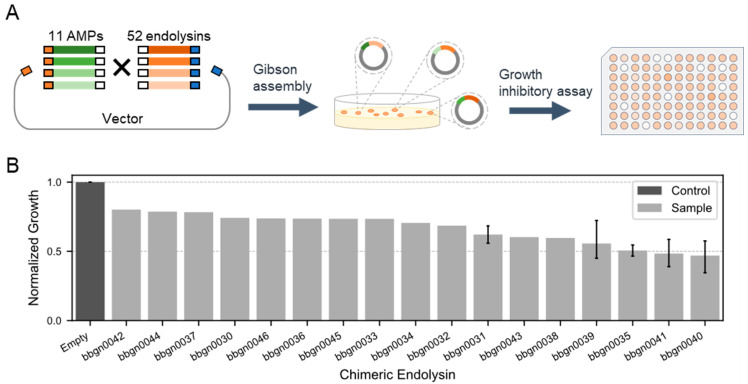
Discovery of chimera AMP–endolysins inhibiting the growth of *A. baumannii.* (**A**) Workflow for the generation of chimeric endolysin inhibiting growth of *A. baumannii*. One endolysin and one AMP are randomly conjugated and recombined into the expression vector by Gibson assembly. (**B**) Growth inhibitory assay against *A. baumannii*. The normalized growth of *A. baumannii* treated by chimeric endolysin in the lysate was calculated from the absorbance of bacterial culture after 16 h incubation. The absorbance of culture treated by lysate of the empty vector recombinant was used as the standard value.

Next, we aimed to construct chimera AMP–endolysins capable of penetrating the outer membrane of Gram-negative bacteria by conjugating AMPs with the 52 natural endolysins (Figure 2A). With reference to previous studies [21,36,37], we selected 11 cecropins, one of AMP, and conjugated them randomly with the 52 endolysins by Gibson assembly, and the gene fragments of chimera AMP–endolysins were cloned into the expression vector. The domain structure of the chimera AMP–endolysins was designed as N’-[AMP]-GAGAGA-[Endolysin]-His6-C’. Through the random assembly reaction, we constructed an expression library comprising up to 572 different chimera AMP–endolysins.

We then evaluated the activity of chimera AMP–endolysins against *A. baumannii*. Lysates were prepared for 744 recombinants from the expression library of chimera AMP–endolysins, and a growth inhibitory assay against *A. baumannii* was conducted. Monitoring the growth of *A. baumannii* treated with lysates based on culture turbidity revealed 37 recombinants expected to be expressing active chimera AMP–endolysins against *A. baumannii*. Compared to the lysate of recombinant containing empty expression vector, the lysate of the 37 recombinants decreased turbidity of the *A. baumannii* culture to less than 80%. Sanger sequencing of the 37 recombinants was performed, and 17 unique chimera AMP–endolysins were found (Table S3). Notably, the top five chimera AMP–endolysins that exhibited relatively strong growth inhibition activity all shared the same AMP sequence (Q86PR4), suggesting the importance of AMP in the antimicrobial activity against Gram-negative bacteria. Eleven chimera AMP–endolysins contained five natural endolysin genes with high lytic activity, and this indicated that not only AMPs but also endolysins contributed to growth inhibition. These results highlighted the significance of both AMP and endolysin combinations in determining the target of antimicrobial activity against Gram-negative bacteria.

### 3.3. Identification of Active Chimeric Endolysin Against A. baumannii

**Figure 3:**
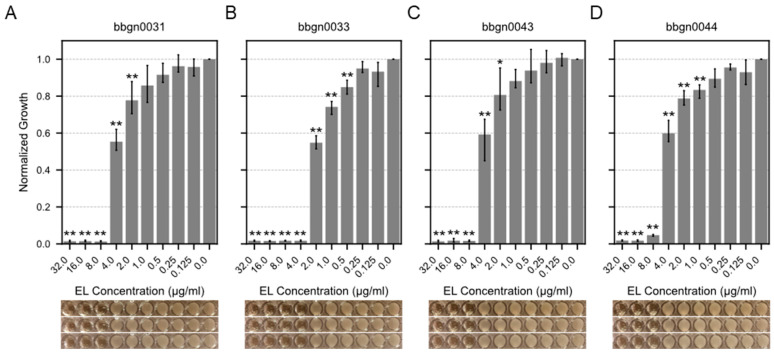
MIC assay of chimera AMP–endolysins. Bar plot showed relative growth of *A. baumannii* treated by chimeric endolysin (**A**) bbgn0031, (**B**) bbgn0033, (**C**) bbgn0043, and (**D**) bbgn0044. The relative growth was calculated from the absorbance of culture. The absorbance of culture which was not treated with endolysin was used as the standard value. Significance was analyzed by the Tukey–Kramer test based on one-way ANOVA and is indicated by asterisks compared to growth of the untreated bacteria (*: *p* < 0.05, **: *p* < 0.01). The image below the graph shows a visual observation of the culture.

We attempted to evaluate the minimum inhibitory concentrations (MIC) of 17 chimera AMP–endolysins against *A. baumannii*. Initially, protein expression conditions were investigated for the 17 chimera AMP–endolysins, and purified proteins of 12 chimera AMP–endolysins were acquired with concentrations exceeding 256 μg/mL, which is required for subsequent assays. Following the broth microdilution method based on the CLSI guideline M07-A9, the MIC value against *A. baumannii* was evaluated for the 12 chimera AMP–endolysins, and four chimera AMP–endolysins (bbgn0031, bbgn0033, bbgn0043, and bbgn0044) demonstrated MIC values ranging from 4 to 8 μg/mL (Figure 3). These MIC values were comparable to similar chimeric endolysin levels reported in previous studies [38,39]. Most of the remaining eight chimera AMP–endolysins also exhibited a growth-reducing effect on *A. baumannii* under the conditions of 32 μg/mL addition, but growth inhibition was not observed within the tested concentration range (Figure S1). Furthermore, a time–kill curve assay of the four chimera AMP–endolysins that showed growth inhibition against *A. baumannii* was also performed, and bbgn0033 exhibited the highest activity, reducing the relative turbidity compared to the control to 70% (Figure S2).

### 3.4. Antibacterial Spectrum of Chimera AMP–Endolysins

To understand the antimicrobial spectrum of the four chimera AMP–endolysins, MIC tests against 10 additional bacterial species were performed (Table 1). No chimera AMP–endolysins showed growth inhibitory activity against the four Gram-positive bacteria (*Staphylococcus aureus*, *Streptococcus gallolyticus*, *Bacillus subtilis*, *Enterococcus faecalis*). When tested against six Gram-negative bacteria (*Escherichia coli*, *Pseudomonas aeruginosa*, *Enterobacter cloacae*, *Klebsiella aerogenes*, *Klebsiella pneumoniae*, *Fusobacterium nucleatum*), three chimera AMP–endolysins (bbgn0031, bbgn0033, and bbgn0043) showed MIC values of 8–16 μg/mL against *P. aeruginosa*. These three endolysins are composed of the same natural endolysin derived from the *Pseudomonas* genome, so their growth inhibitory activity against *P. aeruginosa* was as expected. In addition, bbgn0031 and bbgn0033 also showed MIC values of 16–32 μg/mL against *E. coli*, while bbgn0043 showed no growth inhibitory activity. Both combinations, chimera AMP–endolysins composed of the same natural endolysin and different AMPs (bbgn0031, bbgn0043) and those composed of the different natural endolysin and the same AMPs (bbgn0031, bbgn0043), showed different antimicrobial spectra, suggesting that the appropriate combination of endolysin and AMP is highly important to selective antimicrobial activity.

### 3.5. Cytotoxicity Assay of Chimera AMP–Endolysins

**Figure 4:**
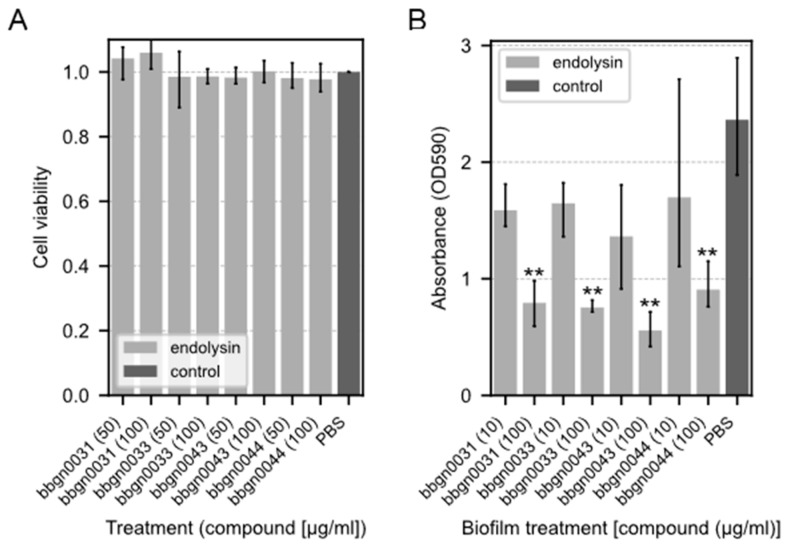
Cytotoxicity assay and biofilm-degrading assay of chimera AMP–endolysins. (**A**) Cytotoxicity assay of chimera AMP–endolysins. The cytotoxicity effect of endolysins was evaluated with the Caco-2 cell line. Relative cell viability was calculated from the absorbance acquired by the CCK-8 assay. The absorbance of the culture treated with PBS was used as the standard value. (**B**) Biofilm-degrading assay of chimera AMP–endolysins with *P. aeruginosa* biofilm. Lower absorbance indicates stronger degradation activity. Both assays were performed with triplicate samples. Significance was analyzed by the Tukey–Kramer test based on one-way ANOVA and is indicated by asterisks compared to the PBS-treated samples (**: *p* < 0.01).

To examine the cytotoxicity of the chimera AMP–endolysins, a cytotoxicity assay using the Cell Counting Kit-8 was performed on the Caco-2 cell line derived from human colon cancer epithelial cells. As a result, no decrease in cell viability was observed in any of the chimeric ELs even after 24 h of treatment with a concentration of 100 μg/mL (Figure 4A), which is a much higher concentration than the MIC against *A. baumannii*.

### 3.6. Biofilm Degradation Assay of Chimera AMP–Endolysins

The biofilm degradation activity of chimera AMP–endolysins was evaluated using *P. aeruginosa*, which is known as one of the drug-resistant bacterial species that form a biofilm. The results showed that all four chimera AMP–endolysins reduced the amount of biofilm to the same extent under the conditions in which they were added (Figure 4B). *P. aeruginosa* biofilm degradation was observed even in bbgn0044, which did not inhibit *P. aeruginosa* growth in the antimicrobial spectral assay.

## 4. Discussion

In this study, we have developed chimera AMP–endolysins with the ability to penetrate the outer membrane of Gram-negative bacteria through the conjugation of endolysins acquired through in silico exploration of Gram-negative bacterial genomes with AMPs. We applied high-throughput screening of hundreds of samples to evaluate chimeric endolysin library containing 500 types of AMP-conjugated endolysins and discovered multiple chimera AMP–endolysins which had antimicrobial activity toward Gram-negative bacteria and different antimicrobial spectra from each other. While many recent studies still find novel endolysins from the isolated phages [40,41,42], our approach successfully identified multiple endolysins with diverse antimicrobial spectra through in silico analysis and the screening process. This represents a significant advancement in the field, as the ability to acquire a broad range of antimicrobial candidates simultaneously accelerates the applicability of the endolysins.

Our data-driven approach was based on the comprehensive genome analysis of both isolated and uncultured bacteria. This approach enables us to search for endolysins from the genomes of closely related species of target bacteria, as demonstrated by the discovery of an *A. junii*-derived endolysin capable of lysing *A. baumannii*. However, in other words, even in data-driven approaches, information about the host bacteria that endolysins and their associated phages infect is important. In recent years, research on phage metagenomics has been increasing, and novel genetic information derived from phages is also being accumulated [43]. However, linking phages to their host organisms remains a significant challenge [44]. The single-cell bacterial genomics may be one of the solutions to accumulating useful phage-derived genetic information because this technology can provide the genes of phages with those of their host bacteria at the same time [26]. Single-cell genomics can also provide genetic information unreached with the metagenomics method, making it a promising tool for uncovering novel sequences, such as the *Pseudomonas*-derived endolysins used in bbgn0031, bbgn0033, and bbgn0043 and showing low similarity to NCBI nr database. Additionally, although the strength of activity varied, the three chimera AMP–endolysins commonly exhibited antimicrobial activity against similar bacterial species. This is consistent with the previous research indicating that the muralytic activity, a key determinant of the antimicrobial spectrum, is driven by the endolysins rather than the AMP [45]. This further highlights the importance of accumulating novel sequences for the development of endolysins with diverse antimicrobial spectra.

Degradation of the *P. aeruginosa* biofilm was observed with bbgn0044 despite its lack of growth inhibition in antimicrobial spectral assays. This activity may be attributed to its specificity for peptidoglycans synthesized during biofilm formation, as *P. aeruginosa* alters peptidoglycan composition between the planktonic and biofilm states [46]. While such specificity can limit broad-spectrum applications, precise control over the endolysin activity offers the potential for a wide range of applications by tailoring them to specific bacterial forms or conditions. With an understanding of target-specific bacterial properties, the strategic development of endolysins with defined targets can maximize their effectiveness and expand their utility across therapeutic and industrial domains.

We developed chimera AMP–endolysins against the target Gram-negative bacteria through the conjugation of natural endolysins and AMPs and evaluated their activity with a high-throughput assay toward 744 samples in a single experiment within a week. In this study, since the theoretical maximum number of chimera AMP–endolysins was 554, we presumed that an assay of several hundred recombinants was sufficient. However, when considering both natural endolysin genes obtained through comprehensive in silico exploration and chimeric endolysin genes obtained through domain shuffling as screening targets, further throughput improvement of the assay is required, as in the previous research [45]. The development of ultra-high-throughput screening technologies, such as microfluidics-based systems, will be critical for exploring the full potential of chimeric libraries [47]. Moreover, refined in silico screening utilizing recent tools like Foldseek is also important for the efficient selection of endolysin candidates [48].

In conclusion, our study highlights the potential of data-driven endolysin development as a powerful approach for discovering multiple endolysins with various antimicrobial spectra. By integrating advances in genomic sequencing, bioinformatics, and screening technologies, this approach is expected to be further enhanced and contribute to the acquisition of useful endolysins.

## Figures and Tables

**Table 1:** Antimicrobial spectra of chimera AMP–endolysins.

Tested Bacteria		bbgn0031	bbgn0033	bbgn0043	bbgn0044
		* chmrAMP	* P81417	* Q963A8	* Q963A8
		** SGM003-00043_05170	** SGM003-00043_05170	** SGM003-00043_05170	** SGM007-00005_02751
Gram-positive	*Staphylococcus aureus*	>32	>32	>32	>32
	*Streptococcus gallolyticus*	>32	>32	>32	>32
	*Bacillus subtilis*	>32	>32	>32	>32
	*Enterococcus faecalis*	>32	>32	>32	>32
Gram-negative	*Escherichia coli*	16	32	>32	>32
	*Acinetobacter baumannii*	8	4	8	8
	*Pseudomonas aeruginosa*	16	8	16	>32
	*Enterobacter cloacae*	>32	>32	>32	>32
	*Klebsiella aerogenes*	>32	>32	>32	>32
	*Klebsiella pneumoniae*	>32	>32	>32	>32
	*Fusobacterium nucleatum*	>32	>32	>32	>32

The values represent MIC (µg/mL) for tested bacterial species. * AMP, ** natural endolysin.

## Data Availability

Amino acid sequences of chimera AMP–endolysins are shown in Table S3.

## Supplementary Materials

The following supporting information can be downloaded at: https://www.mdpi.com/article/10.3390/v17020200/s1, Figure S1: MIC assay of chimeric endolysin candidates against *A. baumannii,* Bar plots showed relative growth of *A. baumannii* treated by chimeric endolysin (A) bbgn0030, (B) bbgn0036, (C) bbgn0038 and (D) bbgn0039 (E) bbgn0040, (F) bbgn0042, (G) bbgn0045 and (H) bbgn0046. The relative growth was calculated from the absorbance of culture. The absorbance of culture which was not treated by endolysin was used as the standard value. Significance was analyzed by the Tukey-Kramer test based on one-way ANOVA and is indicated by asterisks compared to growth of the untreated bacteria (*: *p* < 0.05, **: *p* < 0.01). The image below the graph shows a visual observation of the culture. Figure S2. Time-kill curve of chimeric endolysins against *A. baumannii*: Table S1: Tested candidate endolysins. Table S2: Result of Plate lysis assay. Table S3: Conjugated proteins of chimeric endolysins inhibiting *A. baumannii* growth. Table S4: Bacterial strains used in this study.
