# Supplementary material for: Development of Chimera AMP–Endolysin with Wider Spectra Against Gram-Negative Bacteria Using High-Throughput Assay
Source: Viruses. 2025 Jan 30;17(2):200. doi: 10.3390/v17020200 (PMC11860666; doi:10.3390/v17020200)
Supplement: Supplementary file 1 [file viruses-17-00200-s001.zip › SI_Fig.pdf]

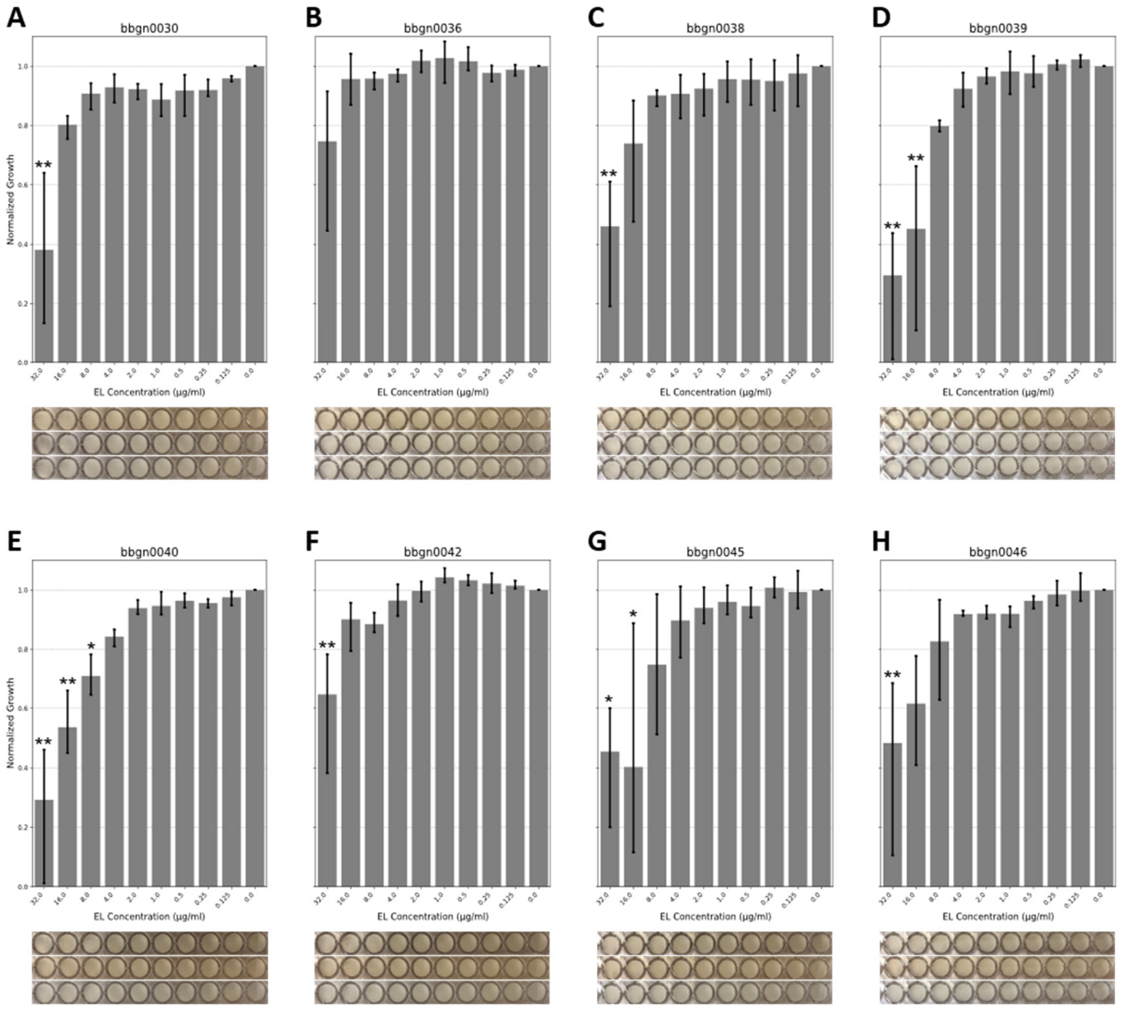

Figure S1. MIC assay of chimeric endolysin candidates against *A. baumannii*

Bar plots showed relative growth of *A. baumannii* treated by chimeric endolysin (A) bbgn0030, (B) bbgn0036, (C) bbgn0038 and (D) bbgn0039 (E) bbgn0040, (F) bbgn0042, (G) bbgn0045 and (H) bbgn0046. The relative growth was calculated from the absorbance of culture. The absorbance of culture which was not treated by endolysin was used as the standard value. Significance was analyzed by the Tukey-Kramer test based on one-way ANOVA and is indicated by asterisks compared to growth of the untreated bacteria (*: $p < 0.05$, **: $p < 0.01$). The image below the graph shows a visual observation of the culture.

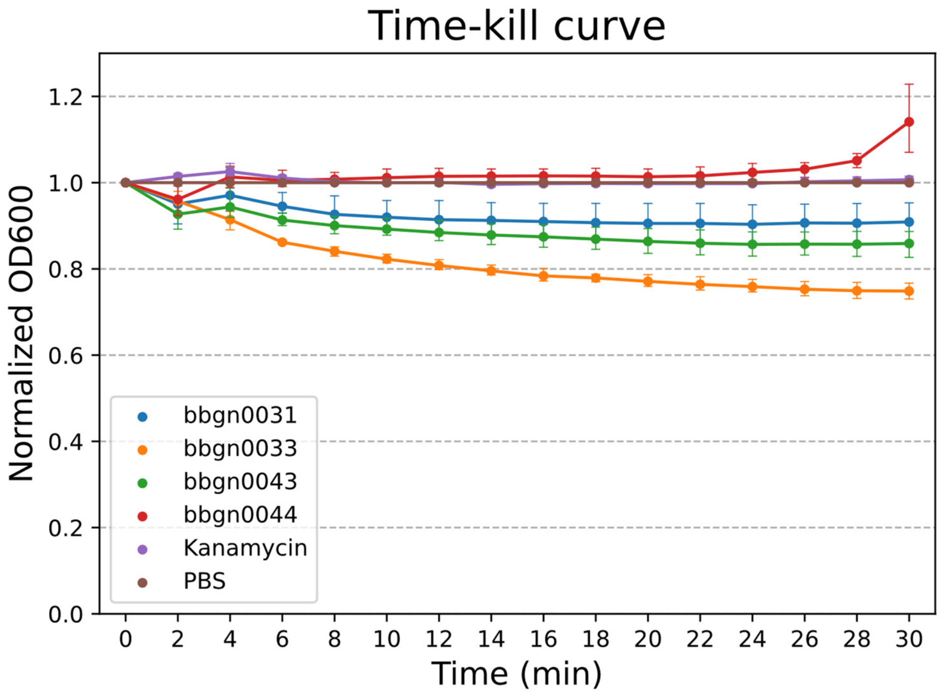

Figure S2. Time-kill curve of chimeric endolysins against *A. baumannii*

Normalized absorbance values were calculated by comparison to the absorbance of the negative control (PBS) at each time point to one.
